# Testing for antiphospholipid antibodies at autopsy

**DOI:** 10.1007/s12024-014-9528-9

**Published:** 2014-01-17

**Authors:** Bartosz Hudzik, Janusz Szkodzinski, Lech Polonski

**Affiliations:** Third Department of Cardiology, Silesian Center for Heart Disease, Medical University of Silesia, Curie-Sklodowska 9, 41-800 Zabrze, Poland

Certain issues require further consideration when testing for antiphospholipid antibodies (APLAs), particularly, when the tests are to be carried out during autopsy [1–3]. First and foremost, laboratory tests must be positive on at least two different occasions, at least 12 weeks apart [4]. We have to keep in mind that APLAs may appear (transiently or persistently) in a wide variety of clinical conditions and even in healthy individuals [5]. Testing for APLAs on only one occasion may lead to false conclusions [1]. Positive results often cause unjustified concern and, conversely, a negative test may provide false reassurance [2].

Furthermore, an appropriate time of testing is one of the most important variables that should always be considered because the acute phase of thrombosis and anticoagulant therapy may considerably affect the results of many assays, making interpretation of the results difficult and unreliable [6, 7]. As a general rule, tests should be postponed for 3–6 months after the acute phase of thrombosis [4–6, 8]. Given that the presence of lupus anticoagulant is determined via a coagulometric assay, there are strict guidelines for its detection. Numerous variables can affect assays used for lupus anticoagulant detection [9, 10]. Optimal laboratory detection of lupus anticoagulant includes proper blood sample collection, the use of appropriate screening, mixing, and confirmatory tests, and proper expression of the obtained results [9]. As the pre-analytical phase (blood collection, double centrifugation, quick sample freezing) is pivotal, we are unaware whether the assays used for lupus anticoagulant detection have been validated for postmortem blood collection and analysis.

Since APS is an acquired condition, testing for APLAs at autopsy for presumed implications for family members seems futile. The condition has scarcely been reported to run in families; however, it does not have a clear pattern of inheritance. Multiple factors (environmental and perhaps genetic) are likely to play a part in the risk of developing APS [5, 11].

There are some reports implicating APLAs in the formation and progression of atherosclerotic lesions [5]. Notwithstanding, as more is learned about the natural history of the development of atherosclerosis, it is clear that the process that results in morbidity and mortality in adults has its origins in childhood and adolescence [12]. So the presence of atherosclerosis in young adults should not be considered as a manifestation of APS, since it is one of the least specific APS clinical pictures. Rather, it would appear that the increasing prevalence of traditional risk factors, such as hypertension, dyslipidemia, smoking, and diabetes mellitus are important in the early stages of the process.

Finally, thrombophilia screening is expensive and time-consuming in clinical practice. It is natural for clinicians to want to look for the cause of thrombosis. However, it is only generally recommended that thrombophilia testing is performed where the management of the patient will be altered by the results, thereby calling into question the ancillary benefits of APS testing at autopsy [2, 13].

## References

[1] Bierton C, Langlois NE (2013). Should we be testing for antiphospholipid antibodies in unexplained pulmonary thromboembolism and atherosclerosis at autopsy?. Forensic Sci Med Pathol.

[2] Machin SJ (2003). Pros and cons of thrombophilia testing: cons. J Thromb Haemost.

[3] Middeldorp S, van Hylckama Vlieg A (2008). Does thrombophilia testing help in the clinical management of patients?. Br J Haematol.

[4] Miyakis S, Lockshin MD, Atsumi T, Branch DW, Brey RL, Cervera R (2006). International consensus statement on an update of the classification criteria for definite antiphospholipid syndrome (APS). J Thromb Haemost.

[5] Khamashta MA (2006). Hughes syndrome: antiphospholipid syndrome.

[6] Margetic S (2010). Diagnostic algorithm for thrombophilia screening. Clin Chem Lab Med.

[7] Heit JA. Thrombophilia: common questions on laboratory assessment and management. Hematology Am Soc Hematol Educ Program. 2007;2007(1):127–35. 10.1182/asheducation-2007.1.127 18024620

[8] Ballard RB, Marques MB (2012). Pathology consultation on the laboratory evaluation of thrombophilia: when, how, and why. Am J Clin Pathol.

[9] Pengo V, Tripodi A, Reber G, Rand JH, Ortel TL, Galli M (2009). Update of the guidelines for lupus anticoagulant detection. Subcommittee on lupus anticoagulant/antiphospholipid antibody of the Scientific and Standardisation Committee of the International Society on Thrombosis and Haemostasis. J Thromb Haemost.

[10] Tripodi A, Moia M, Pengo V. False-negative or false-positive: laboratory diagnosis of lupus anticoagulant at the time of commencement of anticoagulant: a rebuttal. J Thromb Haemost. 2011;9(7):1435–6; author reply 6–7. 10.1111/j.1538-7836.2011.04284.x 21481179

[11] Keeling D, Mackie I, Moore GW, Greer IA, Greaves M (2012). Guidelines on the investigation and management of antiphospholipid syndrome. Br J Haematol.

[12] McGill HC, McMahan CA, Herderick EE, Malcom GT, Tracy RE, Strong JP (2000). Origin of atherosclerosis in childhood and adolescence. Am J Clin Nutr.

[13] Favaloro EJ, McDonald D, Lippi G (2009). Laboratory investigation of thrombophilia: the good, the bad, and the ugly. Semin Thromb Hemost.

